# Prescription medicine use by pedestrians and the risk of injurious road traffic crashes: A case-crossover study

**DOI:** 10.1371/journal.pmed.1002347

**Published:** 2017-07-18

**Authors:** Mélanie Née, Marta Avalos, Audrey Luxcey, Benjamin Contrand, Louis-Rachid Salmi, Annie Fourrier-Réglat, Blandine Gadegbeku, Emmanuel Lagarde, Ludivine Orriols

**Affiliations:** 1 Institut de Santé Publique, d’Epidémiologie et de Développement (ISPED), Université de Bordeaux, Bordeaux, France; 2 Team IETO, Bordeaux Population Health Research Center, UMR U1219, INSERM, Université de Bordeaux, Bordeaux, France; 3 Team Biostatistique, Bordeaux Population Health Research Center, UMR U1219, INSERM, Université Bordeaux, Bordeaux, France; 4 Service d’Information Médicale, Pôle de Santé Publique, Centre Hospitalier Universitaire de Bordeaux, Bordeaux, France; 5 Team Pharmacoepidemiology, Bordeaux Population Health Research Center, UMR U1219, INSERM, Université Bordeaux, Bordeaux, France; 6 Pharmacologie Medicale, Pôle de Santé Publique, Centre Hospitalier Universitaire de Bordeaux, Bordeaux, France; 7 Centre d’Investigation Clinique Bordeaux, INSERM CIC 1401, Centre Hospitalier Universitaire de Bordeaux, Bordeaux, France; 8 Université de Lyon, Lyon, France; 9 UMRESTTE, UMR T9405, l’Institut Français des Sciences et Technologies des Transports, de l’Aménagement et des Réseaux (IFSTTAR), Bron, France; 10 UMRESTTE, Université Lyon 1, Lyon, France; University of Toronto, CANADA

## Abstract

**Background:**

While some medicinal drugs have been found to affect driving ability, no study has investigated whether a relationship exists between these medicines and crashes involving pedestrians. The aim of this study was to explore the association between the use of medicinal drugs and the risk of being involved in a road traffic crash as a pedestrian.

**Methods and findings:**

Data from 3 French nationwide databases were matched. We used the case-crossover design to control for time-invariant factors by using each case as its own control. To perform multivariable analysis and limit false-positive results, we implemented a bootstrap version of Lasso. To avoid the effect of unmeasured time-varying factors, we varied the length of the washout period from 30 to 119 days before the crash. The matching procedure led to the inclusion of 16,458 pedestrians involved in an injurious road traffic crash from 1 July 2005 to 31 December 2011. We found 48 medicine classes with a positive association with the risk of crash, with median odds ratios ranging from 1.12 to 2.98. Among these, benzodiazepines and benzodiazepine-related drugs, antihistamines, and anti-inflammatory and antirheumatic drugs were among the 10 medicines most consumed by the 16,458 pedestrians. Study limitations included slight overrepresentation of pedestrians injured in more severe crashes, lack of information about self-medication and the use of over-the-counter drugs, and lack of data on amount of walking.

**Conclusions:**

Therapeutic classes already identified as impacting the ability to drive, such as benzodiazepines and antihistamines, are also associated with an increased risk of pedestrians being involved in a road traffic crash. This study on pedestrians highlights the necessity of improving awareness of the effect of these medicines on this category of road user.

## Abstract 2

In a case-crossover study, Mélanie Née and colleagues examines the association between use of presciption medicine by pedestrians and risk of injurious road traffic crashes.

## Author summary

**Why was this study done?:**

- Pedestrians account for 22% of the world’s road traffic deaths.
- Medicines have the potential to impair the ability of all road users, including pedestrians.
- To our knowledge, no study so far has investigated the association between consumption of medicinal drugs and risk of road traffic injury as a pedestrian.

**What did the researchers do and find?:**

- We matched French nationwide databases with data on road traffic crashes (collected by police officers) and data on medicine delivery (collected by the national healthcare insurance system).
- We identified 16,458 pedestrians involved in an injurious road traffic crash between 1 July 2005 and 31 December 2011. Among them, 6,584 were included in our analyses.
- Several classes of medicine were associated with an increased risk of a pedestrian being involved in a road traffic crash.
- The most commonly consumed medicines associated with an increased risk of crash included benzodiazepines and benzodiazepine-related drugs, antihistamines, and anti-inflammatory and antirheumatic drugs.

**What do these findings mean?:**

- Increased awareness of the risks of medicine use for pedestrians is important as the risks of medicines in road safety have hitherto been thought to concern drivers only.

## Introduction

Walking is rightly being promoted for its benefits for physical and mental health and for the environment. This comes, however, with a caveat: pedestrians are among the most vulnerable road users [1]. The World Health Organization’s 2013 global status report on road traffic safety revealed that pedestrians account for 22% of the world’s road traffic deaths [2].

A pedestrian crash is defined as any incident occurring on a public thoroughfare that involves at least 1 person on foot and 1 or more vehicles, at least 1 of which is moving. Cognitive, perceptive, and motor skills are involved in a pedestrian’s ability to move safely in a traffic setting. A decrease in motor skills may prevent pedestrians from crossing the road in time. For instance, a study in England compared the walking speed in the population with the speed required to utilize pedestrian crossings among 3,145 adults aged 65 and older and concluded that most of them were unable to cross the road in time [3]. Identified risk factors for the occurrence and severity of injury include environmental factors (e.g., lighting conditions, weather), roadway characteristics (e.g., speed limit, lack of pedestrian facilities), and pedestrian characteristics and behaviors (age, sex, maneuvers, alcohol intoxication) [4–8]. Because medicines have the potential to impair the skills needed to perform road users’ tasks safely, the association between the use of medicines and the risk of road traffic crash has been studied among vehicle drivers. The association between drivers’ use of benzodiazepines and the risk of road crash is now consistently documented [9–14]. Other medicines have also been studied: antidepressants [10,13–17], opioids used in pain treatment and in substitution treatment for opioid dependence [11,13,18,19], and cardiovascular drugs [20,21]. A positive association was found in these studies, except for 1 study [20] that found no association between calcium channel blockers and the risk of crash, and 2 studies [11,19] in which there was an increased risk with opioid use, but the increase was not statistically significant.

Very few studies have investigated the association between the use of medicinal drugs and the risk of road traffic crashes involving pedestrians, and available data remain descriptive, with no comparison group. One study analyzed postmortem blood and urine specimens from victims of road traffic fatalities between 2000 and 2006 in England and Wales. Benzodiazepines and antidepressants were detected in 30% and 20% of pedestrians’ blood and urine specimens, respectively [22]. In 2003, the Australian Transport Safety Bureau reported that 5.4% of male pedestrians aged 15–54 years killed in a road safety crash between 1997 and 1999 had taken benzodiazepine tranquilizers, and 1.3% had taken an antidepressant [23]. The non-comparative design of these studies excludes all conclusions about the actual role of medication use.

The aim of this study was to investigate the association between the use of medicines and the risk of road traffic crash in pedestrians.

## Methods

We extracted and matched data from 3 French nationwide databases: the national healthcare insurance database, police reports, and the police national database of injurious crashes. Drivers were included by means of their national healthcare ID number, extracted from police reports by an automatic procedure. This national ID number was used to link pedestrians to medicine reimbursement data around the crash date. Police reports were matched to records in the injurious crashes database by a probabilistic linkage method [24].

### Ethics statement

Confidentiality was ensured by using the personal information anonymization function of the healthcare insurance system [25]. The study was approved by the French Data Protection Authority.

### Data sources

#### Police reports

French police officers are required to fill out a police report for each injurious crash occurring in the country (about 70,000 reports each year). An injurious crash is defined as a crash occurring in a road open to public circulation involving at least 1 vehicle and resulting in at least 1 victim needing medical attention or being killed. All reports are scanned and stored as image files. A manual validation study of 141 police reports involving pedestrians showed that national ID number was recorded for 39% of the pedestrians involved in these injurious road traffic crashes. These ID numbers were extracted from image files for later matching against dispensing records in the healthcare insurance database. All police reports available from 1 July 2005 to 31 December 2011 were compiled.

#### Police national database of injurious crashes

Information collected by the police for each injurious road traffic crash is stored in the police national database of injurious crashes, including information on the crash, vehicles, and persons involved. The variables used in this study were sex and age of the pedestrian, presumed responsibility for the crash as assessed by police officers, injury severity, weather, season, hour, day of week, lighting conditions, pedestrian action, and pedestrian location.

#### National healthcare insurance database

The healthcare insurance database covers the entire population of France. A record is added each time a reimbursed prescription medicine is dispensed to an outpatient at a pharmacy; the record includes national ID number, date of dispensing, and the 7-digit code that identifies medicines.

### Participant inclusion

A pedestrian was excluded if the police report did not contain his or her national ID number or if the extraction procedure failed or a link could not be established with the corresponding record in the police national database of injurious crashes. If a pedestrian was involved in several crashes during the study period, only the first crash was considered, to ensure that the dispensing of a drug was not a consequence of a previous crash.

### Participant and crash characteristics

Pedestrian characteristics (including age, sex, responsibility attributed by police officers, injury severity, and the pedestrian’s action and location) and crash characteristics (including weather, season, time and day of the crash, and lighting) were described. These characteristics were also used to investigate the factors associated with the probability of being part of the study.

### Medicine and exposure periods

In France, a classification of medicines that could affect the ability to drive, use machines, and implement tasks requiring attention and precision has been developed by the French National Agency for Medicines and Health Products Safety (ANSM) and gradually applied since 2005 [26]. This classification, with its 4-level ranking from 0 (no or negligible risk) to 3 (major risk), has been validated in a study on drivers using the same databases as used in this study [24]. For the present study, only prescription medicines ranking from level 1 to 3 were included in the analysis. These medicines account for 37% of the medicines sold in France [27].

The exposure periods were determined for each pharmacotherapeutic class according to the Anatomical Therapeutic Chemical (ATC) classification (fourth level). Medication exposure was defined as starting 1 day after dispensing, and exposure duration was estimated from median values reported in a survey on medicine prescription in France [28]. To ensure that the medicines were not prescribed as a consequence of injuries sustained in the crash, medicines dispensed on the day of the crash were not considered in the analysis.

### Case-crossover analysis

The case-crossover design allows study of the effect of transient exposure on the risk of acute events. The exposure frequency during a period just before the crash (case period) is compared with the exposure frequency during an earlier period (control period) in the same individual [29]. In the case-crossover analysis, only records with discordant exposures in case and control windows (either exposed on the case day and unexposed on the control day or unexposed on the case day and exposed on the control day) contribute to the analysis. The period between the case and control periods is called the washout. It corresponds to the period needed to ensure that exposure in the control period is not mixed with exposure in the case period.

**Fig 1:**
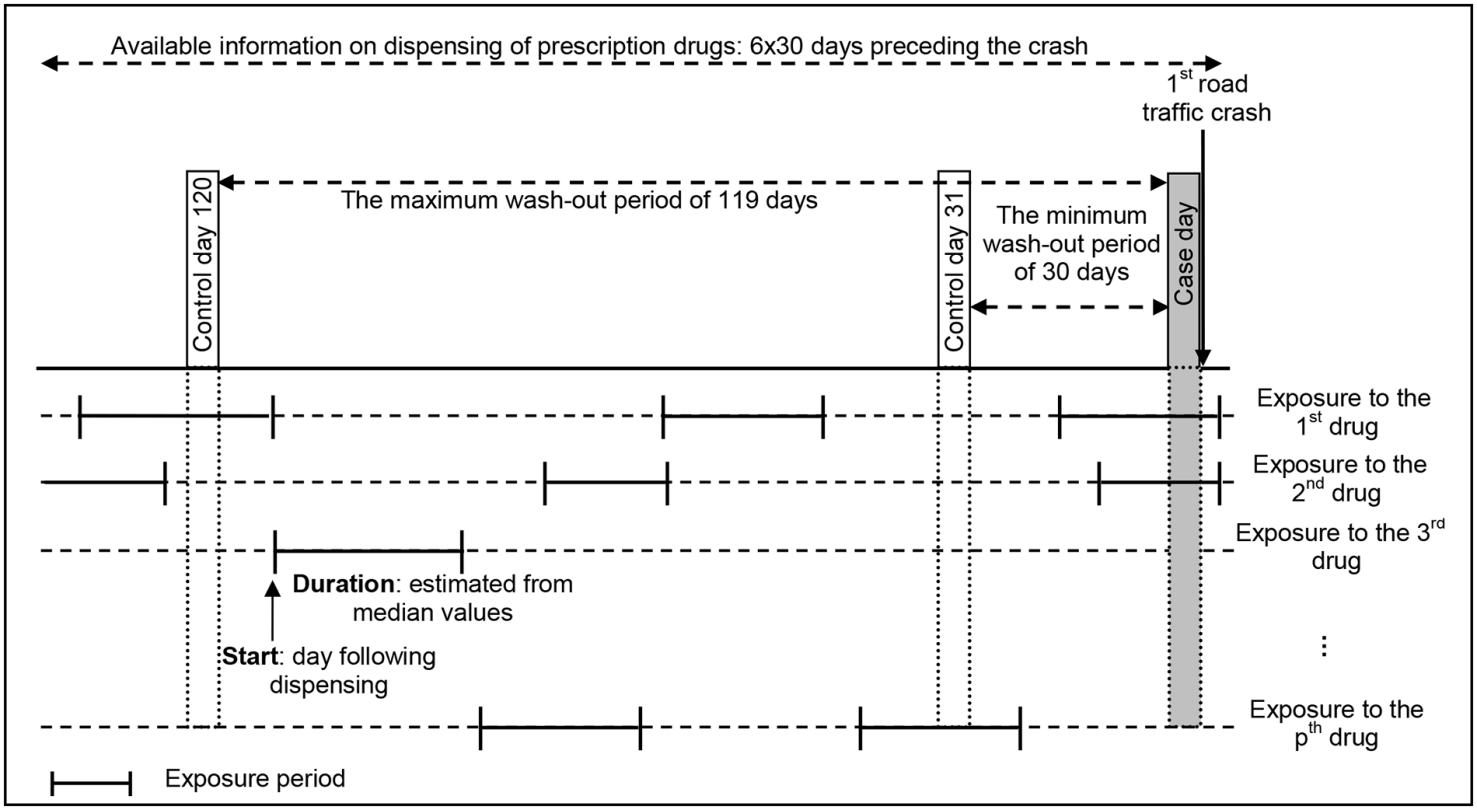
The case-crossover design of the study, with multiple drug exposures and a varying washout period. In the case-crossover design, only individuals with unequal exposures for the control period and the case period contribute to the analysis. For instance, with a control day defined at 120 days before the crash day, the individual shown in this figure has unequal exposures for the second drug (exposed during case day and unexposed during control day), but concordant exposures for the first drug (exposed both days) and the third drug (unexposed both days). In a case-crossover analysis, only the exposure to the second drug is used.

The optimal washout period for a given medicine depends on its indication and prescription patterns. Since all prescribed drugs with non-null risk level were considered here, we could not make any assumption concerning the choice of the washout period. This period was thus varied from 30 to 119 days before from the crash day, resulting in the implementation of 90 case-crossover designs with distinct washout periods (Fig 1). Data on reimbursed medicines dispensed within 6 months before the crash were available. To ensure that the pedestrian was not exposed to a medicine at the beginning of the observation period without us knowing (dispensing date before the beginning of the observation period), we chose a 4-month study period. This approach of using multiple washout periods provided an indication of the robustness of the observed associations.

The conditional logistic regression model was the standard tool used for the analysis.

### Lasso analysis

We designed the analysis protocol assuming that few medicines increased the risk of road crash. A variable selection procedure thus had to be performed to identify these medicines from a large list of potential candidates. To address multiple testing problems, we implemented a Lasso (least absolute shrinkage and selection operator) regression analysis [30] for each washout period. In this multivariable modeling approach, a penalty term shrinks each coefficient estimate and sets some of them to zero. Estimation and selection of the subset of covariates showing the strongest association from a longer list of covariates occur simultaneously.

The Lasso approach tends to retain not only the relevant exposures but also a few additional irrelevant ones (though typically their estimates are small). To circumvent this problem, we used the Bolasso (bootstrap-enhanced least absolute shrinkage operator) method [31]. Only exposures frequently chosen by the Lasso analysis over the bootstrap samples were selected, which improved the stability of the results [32]. In the Bolasso procedure, 2 parameters have to be tuned. The optimal amount of shrinkage was estimated by cross-validation. The frequency threshold was tuned with the Akaike information criterion over 1,000 bootstrap samples. To correct bias in the estimated coefficients, we fitted the unpenalized logistic regression model with the exposures retained in the model (those having a nonzero point estimate of log-odds ratio) [33].

Results for each of the 90 control periods were combined in a figure to investigate whether patterns could be found according to the length of the washout period (e.g., medicines selected with narrow washout). A colored square was used to indicate that a medicine class was selected by the model. The color intensity and the size of this square were proportional to the bias-corrected odds ratio obtained at the end of the Bolasso procedure.

All statistical analyses were performed using the package Penalized in R statistical software (version 3.2.3; R Foundation for Statistical Computing, Vienna, Austria). The bootstrap was implemented manually.

## Results

National ID number, sex, and date of birth of individuals involved in a traffic crash were extracted for 215,032 of the 439,518 police reports of traffic crashes from 1 July 2005 to 31 December 2011. Among these, 186,636 (86.8%) were matched with a corresponding record in the police national database of injurious crashes. The linkage failed for national ID numbers corresponding to road users involved in a crash but not captured in the police national injurious crashes database and for individuals not involved in the crash (e.g., witnesses).

**Fig 2:**
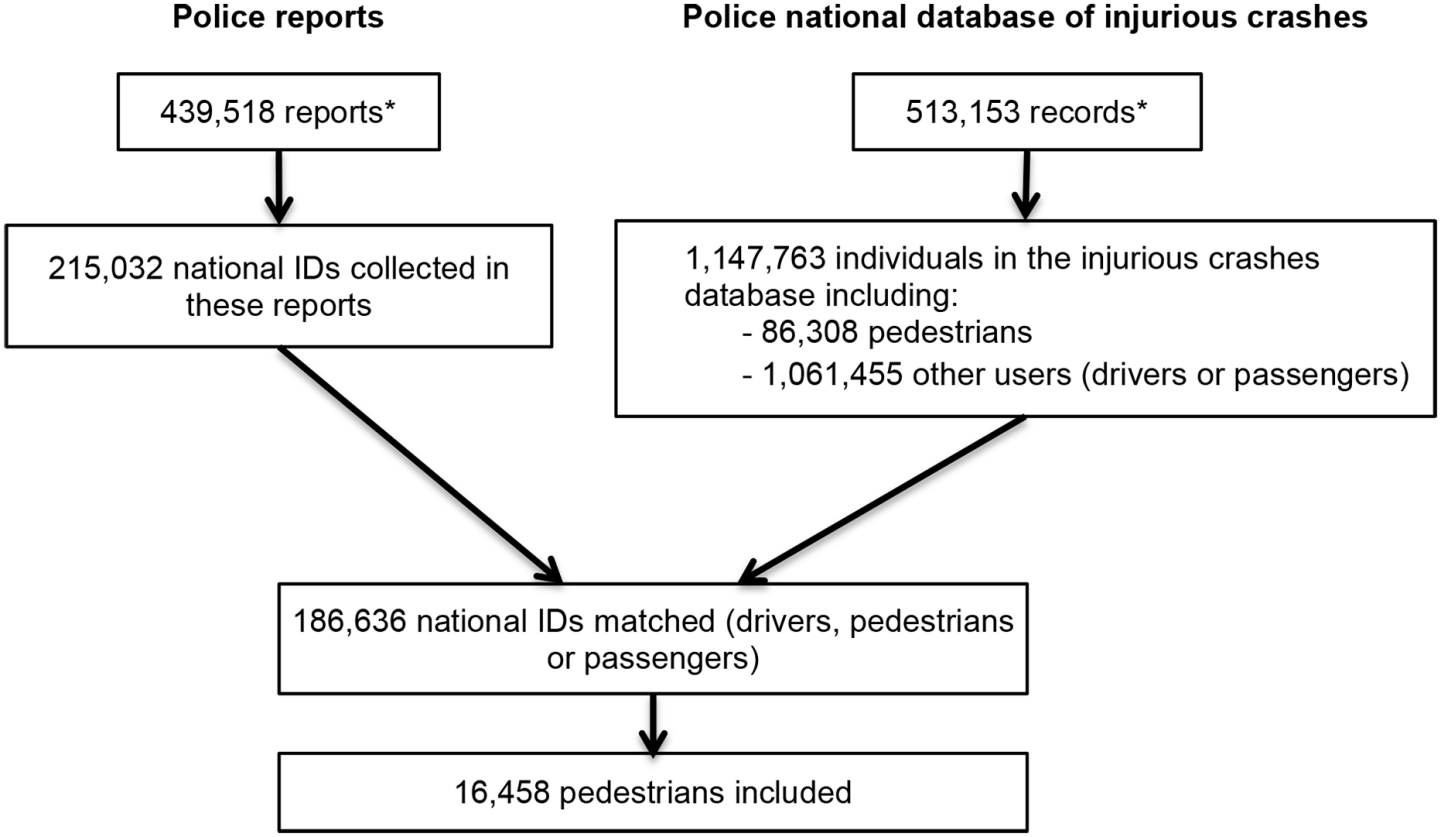
Flowchart of the inclusion procedure. Note that the discrepancy between the number of police reports and the number of records in the police national database of injurious crashes is explained by the fact that a small proportion of unavailable reports were being used for ongoing legal investigations. *Modified from Orriols et al. [24].

The procedure led to the inclusion of 16,458 pedestrians involved in a road traffic crash between 1 July 2005 and 31 December 2011 (19.1% of the pedestrians registered in the national database of injurious crashes; see Fig 2). Among these, 7,535 pedestrians (45.8%) were never exposed to any medicine under study during the time period considered (crash day and 120 days before), and 2,339 pedestrians (14.2%) were exposed to at least 1 medicine of interest, but without interruption. Consequently, these pedestrians were not included in the analyses. When varying the washout period, the sample size (pedestrians with unequal exposure in the case and control periods) varied from 5,009 (30.4% of pedestrians; washout = 37 days) to 5,315 (32.3% of pedestrians; washout = 107 days). The probability of being part of the study was associated with sex, age, injury severity, weather conditions, and day of the week (S1 Table).

Almost 60% of the participants were women, and almost one-third were 65 years old or older (Table 1). The majority of crashes occurred during favorable weather and lighting conditions. In all, 12,474 pedestrians (75.8%) were crossing the street at the time of the crash, and 7,823 (47.6%) were in a crosswalk. More precisely, most of the crashes occurred in a crosswalk with a traffic light (29.4%) or less than 50 m from a crosswalk (20.8%).

**Table 1:** Pedestrian and crash characteristics.

Characteristic	Subcategory	Number (%)
**Total**		16,458
**Gender male**		6,803 (41.3)
**Age (years)**	<18	1,455 (8.8)
	18–24	1,641 (10.0)
	25–34	1,731 (10.5)
	35–44	1,810 (11.0)
	45–54	2,122 (12.9)
	55–64	2,255 (13.7)
	65–74	2,022 (12.3)
	≥75	3,395 (20.6)
	Missing	27 (0.2)
**Pedestrian responsible for crash (as determined by police officer)**		2,369 (14.4)
**Injury severity**	Unhurt	123 (0.7)
	Slightly injured	8,197 (49.8)
	Seriously injured	7,741 (47.0)
	Killed	397 (2.4)
**Risk ranking of medicine**^a^	Level 1	3,460 (21.0)
	Level 2	3,379 (20.5)
	Level 3	983 (6.0)
**Weather**	Not reported	1 (0.0)
	Normal	12,844 (78.0)
	Light rain	1,852 (11.2)
	Heavy rain	473 (2.9)
	Snow/hail	118 (0.7)
	Fog/smoke	76 (0.5)
	Strong windstorm	34 (0.2)
	Blinding weather	381 (2.3)
	Cloudy	572 (3.5)
	Other	107 (0.7)
**Season**	Spring	3,289 (20.0)
	Summer	3,475 (21.1)
	Autumn	5,601 (34.0)
	Winter	4,093 (24.9)
**Time of day**	05:00–10:59	4,464 (27.1)
	11:00–13:59	2,865 (17.4)
	14:00–19:59	7,762 (47.2)
	20:00–22:59	923 (5.6)
	23:00–01:59	286 (1.7)
	02:00–04:59	158 (1.0)
**Crash day**	Weekday	13,500 (82.0)
	Saturday	1,958 (11.9)
	Sunday	1,000 (6.1)
**Lighting**	Daylight	12,028 (73.1)
	Dawn or dusk	1,007 (6.1)
	Dark, no street lights	498 (3.0)
	Dark, street lights off	132 (0.8)
	Dark, street lights on	2,793 (17.0)
**Pedestrian’s action**	Not reported	715 (4.3)
	Moving	1,340 (8.1)
	Crossing	12,474 (75.8)
	Playing/running	470 (2.9)
	Other	1,459 (8.9)
**Pedestrian’s location**	Not reported	1,632 (9.9)
	>50 m from a crosswalk	2,064 (12.5)
	≤50 m from a crosswalk	3,416 (20.8)
	Crosswalk without a traffic light	2,990 (18.2)
	Crosswalk with a traffic light	4,833 (29.4)
	Sidewalk	1,028 (6.2)
	Other	495 (3.0)

^a^Exposure on the crash day. For this study, only medicines ranking from levels 1 to 3 were included in the analysis. Some pedestrians may have been exposed to several medicines of different risk level.

**Fig 3:**
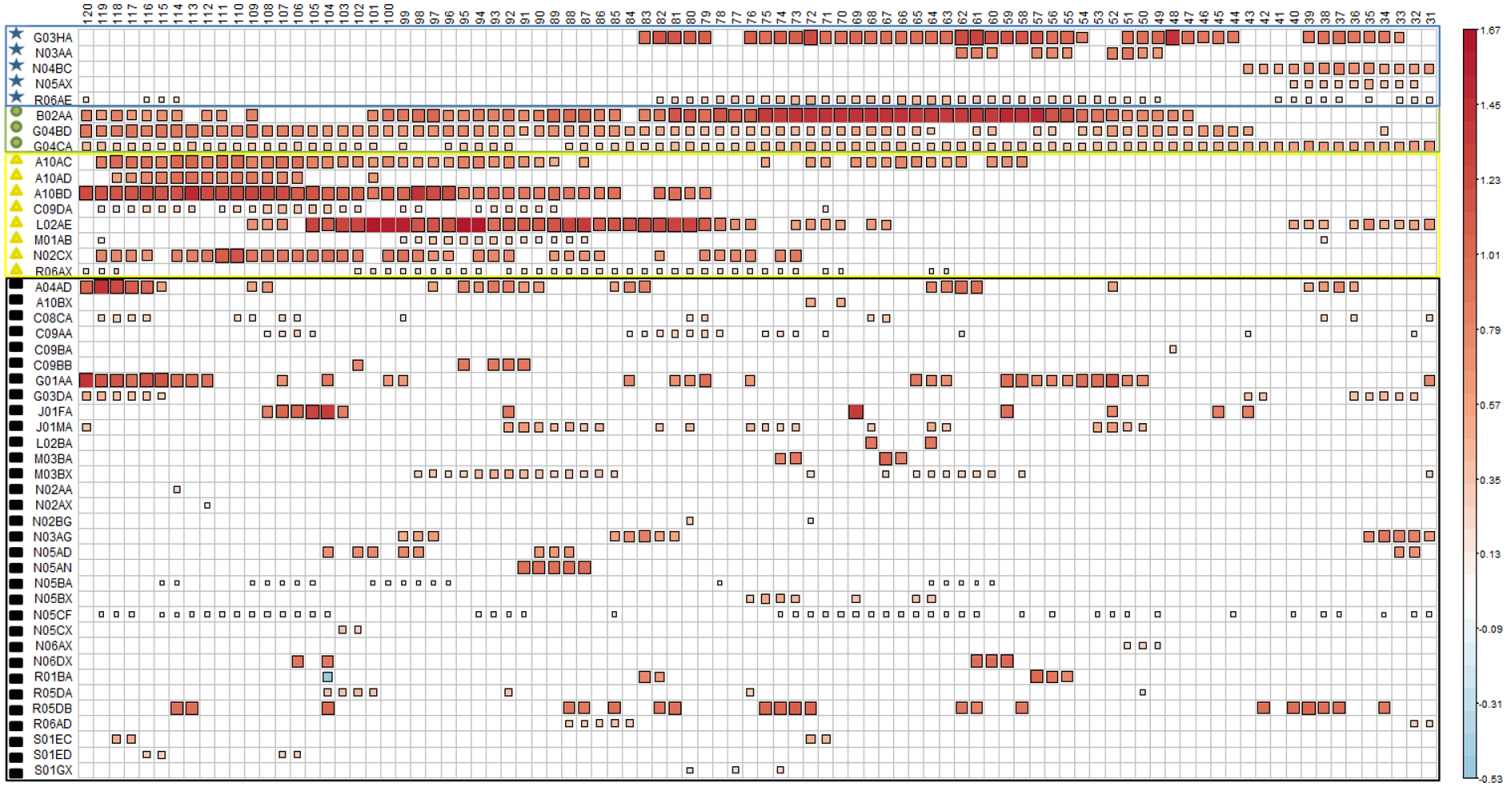
Results of the 90 case-crossover designs obtained when varying the washout period from 30 days to 119 days. A blank cell means that the medicine class was not retained in the final model for this control period, and a colored square means that the medicine class was selected by the model. Both the size and color intensity of the squares depend on the absolute value of the bias-corrected estimated coefficients. When varying the washout period, the frequency thresholds estimated using the Akaike information criterion varied from a minimum of 50% (washout = 40) to a maximum of 74% (washout = 104). A frequency threshold of 74% means that medicines selected in at least 74% of the 1,000 bootstraps were considered as associated risk factors for pedestrian road crash. The different colored forms on the far left indicate groups of medicines according to the location of the control periods (with respect to the crash day) for which there was an association of the medicine with increased risk of being involved in a road crash as a pedestrian: blue stars indicate increased risk in control periods close to the crash; yellow squares indicate increased risk in control periods far from the crash; green circles indicate increased risk in control periods both close to and far from the crash; black squares indicate increased risk in discontinuous control periods.

When varying the washout period from 30 days (Fig 3, right) to 119 days (Fig 3, left), we found 48 medicine classes (ATC level 4) associated with an increased risk of being involved in a road crash as a pedestrian in at least 1 of the 90 models, and 4 association patterns (Fig 3). A pattern with a narrow washout period characterized 5 medicine classes (labeled with blue stars in Fig 3), including dopamine antagonists (N04BC) and piperazine derivatives (R06AE). A second pattern included 8 medicines that were consistently associated with increased risk in models with a wide washout period (yellow triangles). This pattern included 3 insulins and analogues (A10AC, A10AD, and A10BD). A third pattern included 3 medicine classes that were regularly associated with increased risk regardless of the washout period (green circles). Among these 3, alpha-adrenoreceptor antagonists (G04CA) showed the most regular association profile (showing association with increased risk in more than 90% of the designs). Finally, 32 medicines showed irregular associations with pedestrian crash risk (black squares) with respect to the length of the washout period.

Table 2 presents the variation of the bias-corrected odds ratios and the total number of designs in which each exposure was selected (*L* value).

**Table 2:** Selection frequency of 48 medicine classes among the 90 case-crossover models, bias-corrected odds ratios, and unequal exposures.

ATC class^a^ (fourth level)	ATC class description	*L*^b^	OR^c^	Case period^d^	Control period^e^
G03HA*	Antiandrogens, plain	43	2.66 (2.59–3.10)	10 (9–10)	4 (3–4)
N03AA*	Barbiturates and derivatives	10	2.16 (2.00–2.30)	13 (13–13)	6 (6–7)
N04BC*	Dopamine agonists	13	1.85 (1.80–1.99)	22 (20–23)	12 (11–12)
N05AX*	Other antipsychotics	9	1.48 (1.45–1.55)	45 (44–45)	31 (30–32)
R06AE*	Piperazine derivatives	47	1.35 (1.28–1.49)	122 (113–126)	85 (82–88)
B02AA^#^	Amino acids	64	2.60 (2.23–3.93)	13 (12–13)	4 (3–5)
G04BD^#^	Urinary antispasmodics	74	1.87 (1.61–2.01)	42 (40–44)	22 (21–25)
G04CA^#^	Alpha-adrenoreceptor antagonists	84	1.50 (1.44–1.61)	59 (56–63)	39 (36–42)
A10AC^†^	Insulins and analogues for injection, intermediate-acting	46	2.10 (1.88–2.33)	18 (16–18)	9 (7–9)
A10AD^†^	Insulins and analogues for injection, intermediate-acting combined with fast-acting	14	2.30 (2.15–2.41)	20 (18–21)	9 (8–9)
A10BD^†^	Combinations of oral blood glucose–lowering drugs	40	2.89 (2.49–3.40)	11 (10–13)	4 (4–4)
C09DA^†^	Angiotensin II antagonists and diuretics	26	1.34 (1.28–1.38)	102 (95–107)	74 (70–79)
L02AE^†^	Gonadotropin-releasing hormone analogues	48	2.98 (1.87–3.44)	13 (11–18)	4 (3–10)
M01AB^†^	Acetic acid derivatives and related substances	15	1.30 (1.27–1.39)	90 (89–94)	68 (64–70)
N02CX^†^	Other antimigraine preparations	36	2.21 (2.09–2.44)	13 (12–13)	6 (5–6)
R06AX^†^	Other antihistamines for systemic use	36	1.20 (1.18–1.23)	209 (206–213)	172 (166–176)
A04AD^‡^	Other antiemetics	27	2.16 (1.84–2.53)	18 (18–19)	9 (7–10)
A10BX^‡^	Other blood glucose–lowering drugs, excluding insulins	2	1.69 (1.69–1.69)	19 (19–19)	11 (11–11)
C08CA^‡^	Dihydropyridine derivatives	16	1.32 (1.30–1.35)	81 (75–83)	61 (56–62)
C09AA^‡^	ACE inhibitors, plain	18	1.26 (1.23–1.28)	125 (120–128)	96 (95–100)
C09BA^‡^	ACE inhibitors and diuretics	1	1.37 (1.37–1.37)	48 (48–48)	36 (36–36)
C09BB^‡^	ACE inhibitors and calcium channel blockers	5	2.45 (2.41–2.45)	9 (9–9)	4 (4–4)
G01AA^‡^	Antibiotics	32	2.44 (2.10–2.58)	13 (13–13)	5 (5–6)
G03DA^‡^	Pregnen (4) derivatives	13	1.46 (1.42–1.49)	44 (43–45)	30 (28–31)
J01FA^‡^	Macrolides	12	2.55 (2.30–2.98)	11 (11–11)	4 (4–5)
J01MA^‡^	Fluoroquinolones	21	1.54 (1.42–1.61)	45 (44–45)	28 (27–29)
L02BA^‡^	Anti-estrogens	2	2.48 (2.46–2.49)	8 (8–8)	3 (3–3)
M03BA^‡^	Carbamic acid esters	4	2.42 (2.30–2.57)	14 (13–14)	6 (5–6)
M03BX^‡^	Other centrally acting agents	24	1.37 (1.33–1.43)	70 (67–71)	50 (47–52)
N02AA^‡^	Natural opium alkaloids	1	1.24 (1.24–1.24)	112 (112–112)	89 (89–89)
N02AX^‡^	Other opioids	1	1.16 (1.16–1.16)	214 (214–214)	184 (184–184)
N02BG^‡^	Other analgesics and antipyretics	2	1.31 (1.30–1.32)	77 (76–78)	60 (60–60)
N03AG^‡^	Fatty acid derivatives	13	1.90 (1.80–2.25)	22 (21–23)	11 (10–12)
N05AD^‡^	Butyrophenone derivatives	10	2.01 (1.97–2.04)	16 (16–17)	10 (9–11)
N05AN^‡^	Lithium	5	2.68 (2.67–2.69)	8 (8–8)	3 (3–3)
N05BA^‡^	Benzodiazepine derivatives	19	1.12 (1.11–1.13)	462 (437–470)	411 (391–413)
N05BX^‡^	Other anxiolytics	7	1.49 (1.43–1.57)	37 (37–38)	26 (25–26)
N05CF^‡^	Benzodiazepine-related drugs	47	1.17 (1.16–1.20)	251 (238–256)	212 (200–217)
N05CX^‡^	Hypnotics and sedatives in combination, excluding barbiturates	2	1.40 (1.39–1.41)	54 (54–54)	37 (37–37)
N06AX^‡^	Other antidepressants	3	1.28 (1.26–1.30)	112 (111–112)	89 (89–89)
N06DX^‡^	Other antidementia drugs	5	2.68 (2.52–2.69)	8 (8–8)	3 (3–3)
R01BA^‡^	Sympathomimetics	6	2.16 (1.91–2.38)	15 (15–15)	7 (6–7)
R05DA^‡^	Opium alkaloids and derivatives	7	1.44 (1.39–1.45)	59 (59–59)	42 (41–45)
R05DB^‡^	Other cough suppressants	21	2.66 (2.51–2.76)	8 (8–8)	3 (3–3)
R06AD^‡^	Phenothiazine derivatives	7	1.38 (1.35–1.39)	67 (67–67)	50 (48–50)
S01EC^‡^	Carbonic anhydrase inhibitors	4	1.60 (1.60–1.61)	25 (25–25)	16 (15–16)
S01ED^‡^	Beta-blocking agents	4	1.32 (1.31–1.34)	64 (63–64)	48 (47–48)
S01GX^‡^	Other antiallergics	3	1.28 (1.28–1.29)	83 (83–83)	61 (61–61)

^a^ATC: the Anatomical Therapeutic Chemical classification system.

^b^L: number of case-crossover designs in which the exposure was selected.

^c^OR: median, first quartile, and third quartile of bias-corrected odds ratio.

^d^Case period: median, first quartile, and third quartile of the number of cases exposed only during the case period.

^e^Control period: median, first quartile, and third quartile of the number of cases exposed only during the control period.

*ATC classes with risk associations for control periods close to the crash day (blue stars in Fig 3).

^#^ATC classes with risk associations for control periods both close to and far from the crash day (green circles in Fig 3).

^†^ATC classes with risk associations for control periods far from the crash day (yellow triangles in Fig 3).

^‡^ATC classes with risk associations for discontinuous control periods (black squares in Fig 3).

Table 3 shows that benzodiazepine derivatives and benzodiazepine-related drugs have the highest consumption rates in our cohort. Medicines with frequent use also include 4 classes of drugs used either as analgesics or as anti-inflammatory and antirheumatic drugs (R05DA, N02BG, N02AX, and M01AB), 2 classes of antihistamines (R06AX and R06AD), plain ACE inhibitors (C09AA), and 1 class of antidepressants (N06AX).

**Table 3:** The 10 most consumed medicines among those listed in Table 2 as associated with road traffic crash involvement.

ATC^a^ class (fourth level)		Number (%)^b^	*L*^c^
Total		16,458	
N05BA^‡^	Benzodiazepine derivatives	1,986 (12.07)	19
N05CF^‡^	Benzodiazepine-related drugs	1,004 (6.10)	47
R05DA^‡^	Opium alkaloids and derivatives	787 (4.78)	7
N02BG^‡^	Other analgesics and antipyretics	766 (4.65)	2
N02AX^‡^	Other opioids	752 (4.57)	1
R06AX^†^	Other antihistamines for systemic use	743 (4.51)	36
C09AA^‡^	ACE inhibitors, plain	687 (4.17)	18
M01AB^†^	Acetic acid derivatives and related substances	599 (3.64)	15
N06AX^‡^	Other antidepressants	536 (3.26)	3
R06AD^‡^	Phenothiazine derivatives	506 (3.07)	7

^a^ATC: the Anatomical Therapeutic Chemical classification system.

^b^Frequency and proportion of individuals with at least 1 delivery over the study period.

^c^L: number of case-crossover designs in which the exposure was selected.

^‡^ATC classes with risk associations for discontinuous control periods (black squares in Fig 3).

^†^ATC classes with risk associations for control periods far from the crash (yellow triangles in Fig 3).

## Discussion

The analysis of 16,458 pedestrians involved in a road crash between 1 July 2005 and 31 December 2011, among which 6,584 were included in the analyses, identified 48 medicine classes associated with an increased risk for pedestrians of being involved in an injurious road traffic crash. Among them, the 10 most consumed medicines included benzodiazepines and benzodiazepine-related drugs, antihistamines, and anti-inflammatory and antirheumatic drugs.

Injury severity and age were associated with the probability of being part of the study. This is due to 2 combined factors: (1) the healthcare number was more frequently noted for injured pedestrians admitted to hospital, leading to a slight overrepresentation of pedestrians injured in more severe crashes, and (2) older road users involved in a road crash are more likely to be more severely injured [34].

Computerized records of reimbursed prescriptions were used to determine exposure to medications. Compared with self-reporting methods, this method avoids memory bias. However, these databases have 2 main limitations. First, there is a lack of information about self-medication and the use of over-the-counter drugs because only dispensing of reimbursed drugs is recorded. Second, there is a lack of information about treatment compliance. Because of the use of the dispensing date to determine exposure periods, these limitations can result in exposure misclassification. A study using the same reimbursement database showed that healthcare insurance data are reliable indicators of actual exposure for medicines used over a long time, but less so for episodically used medicines [35].

In studies on medicines, both the treatment and the condition for which the treatment was prescribed can be associated with the outcome of interest. Because the case-crossover design compares each individual with himself/herself, it has the advantage of eliminating control selection bias and confounding by time-invariant factors such as age, sex, and chronic conditions [36]. However, confounding by indication remains for non-chronic conditions or if there is a change in the severity of the disease over time. Confounding may also remain for other time-varying factors such as increased enforcement of speeding laws. However, the effect on our estimates is likely insignificant because exposures of only the 4 months preceding the crash day were considered for each pedestrian. The probability of such an event occurring during these 4 months is therefore small. Another reason the effect of these potential confounding factors is likely to be insignificant is that these 4-month periods were different for every pedestrian and therefore were spread over a 6-year period. The choice of the control periods has to be independent of exposure, and only periods in which an event can occur must be considered [37].

One limitation of our study is that no information was available on how much the participants walked during the control versus case periods. Thus, we were not able to control for the amount of walking. If the probability of being a pedestrian is correlated with exposure, then the results may be biased. Indeed, consumption of some of the medicines under study may result in an increased probability of walking instead of driving because of the warning pictogram on medicine packaging. However, a recent study exploring trends in medicine consumption (restricted to hypnotics and anxiolytics) before and after the implementation of pictograms in France showed no impact of pictograms on exposure level to these medicines among drivers. Consequently, it is very unlikely that the pictogram system resulted in a substantial shift in transportation means for consumers of those medicines [38]. Medicines can also lead to an increase in walking due to an increased sense of well-being. However, there will be a bias only if the reason for taking this medicine already existed during the control period and led to a decrease in walking. This is unlikely, as control periods were at least 30 days before the crash, and there is no reason to think that people affected by a debilitating disease for at least 1 month would not take medicines. Consequently, taking medicines during the case period was assumed to enable the individual to get back to his or her previous walking habits.

Analyses were not adjusted for alcohol consumption because the information was not available during control periods. Alcohol has been found to be associated with a higher risk of crash for pedestrians [39] and could also be a confounding factor for medicines such as benzodiazepines. Information about blood alcohol level, as recorded in the police national database of injurious crashes, was unknown for 41.6% of the pedestrians. Among the 9,617 pedestrians with data for blood alcohol level, 95.7% had a level below the legal limit. A comparison between pedestrians with an alcohol level below the legal limit and pedestrians with missing alcohol status suggested that the latter were not tested for alcohol because the police officers did not consider it necessary. Pedestrians with a blood alcohol level above the legal limit were more likely to be male, aged 30 to 49 years, and presumed responsible for the crash, compared with pedestrians with an alcohol level below the legal limit or with missing alcohol status. They were also less likely to be exposed to level 1 medicines and more likely to be exposed to level 3 medicines. Removing the individuals with an alcohol level above the legal limit from the analysis would most likely be inconsequential because they represent only 412 individuals, thus resulting in a very small number of discordant exposures.

Among the 16,458 pedestrians included in the present study, older adults and women were overrepresented. These findings are in agreement with national statistics on injurious crashes [40]. This is probably the combined result of a greater fragility and exposure of the elderly, along with a higher proportion of women among the elderly. Two urological drugs (G04CA and G04BD) were selected for almost all case-crossover designs regardless of the washout period. This can be interpreted as the result of treatment initiation (they were exposed during the case period, but never before). These drugs can affect a pedestrian’s level of attention (e.g., reaction time) or mobility (e.g., walking speed). For instance, alpha-adrenoceptor antagonists induce vasodilatation and subsequently lower blood pressure, with potential symptoms including asthenia, dizziness, fatigue, and orthostatic hypotension, particularly upon treatment initiation [41–43]. Similarly, urinary antispasmodics have a high potential of producing anticholinergic effects, which can result in cognitive side effects including memory changes, blurred vision, somnolence, hallucinations, confusion, and delirium [44,45].

Eight medicines (ATC level 4) were selected in case-crossover designs with control periods away from the crash day. Risk associations with long washout periods may be the consequence of longer duration of medicine use, requiring a longer washout period in order to have discordant exposures between case and control periods. The exposures selected in this profile, including antidiabetic drugs (A10AC, A10AD, and A10BD) and antimigraine preparations (N02CX) prescribed in chronic treatment of migraine, tend to support this assumption. Hypoglycemia is a common adverse effect of antidiabetic treatment [46–48] and can result in decreased motor skills, visual acuity, or auditory processing and deterioration of a variety of cognitive processes [49,50]. However, in this pattern, the increased risk is most likely linked to disease-related complications. Indeed, in the case-crossover design, the longer the washout period, the higher the risk of potential confounding by unmeasured time-varying factors including disease severity. Medicines of the R06AX group were mostly second-generation antihistamines, which may have fewer sedative effects than those of the first generation. Rhinitis, for which they are prescribed, can affect the quantity and quality of sleep and so lead to fatigue and daytime somnolence [51,52]. Acetic acid derivatives and related substances (M01AB) are nonsteroidal anti-inflammatory drugs used in the treatment of various painful inflammatory conditions including rheumatoid arthritis and osteoarthritis. Rheumatoid arthritis, which may affect pedestrians’ walking ability, is characterized in most people by alternating periods of remission and relapse, which can explain the narrow washout despite it being a chronic condition [53]. Antihistamines (R06AX) and acetic acid derivatives (M01AB) were among the 10 most consumed medicines selected in our analyses.

Risk associations with exposure periods close to the crash suggest an effect of acute, short-term exposure to the medicine. Both Parkinson disease symptoms (e.g., muscle rigidity, slowing of physical movement) and side effects of dopamine agonists (N04BC)—including dizziness, somnolence, insomnia, and orthostatic hypotension—can affect a pedestrian’s walking ability. Because of the chronicity of Parkinson treatment, the increased risk of crash can be the result of a treatment change or a change in the disease condition. However, because of the proximity with the crash, the first possibility is more likely. This is also true for other antipsychotic drugs (N05AX) and barbiturates and derivatives (N03AA), 2 medicines used in chronic conditions. Moreover, lack of compliance with treatment is frequent in patients with schizophrenia, and barbiturates and derivatives (N03AA) have been found to be associated with drowsiness and cognitive effects, including diminished attention, executive function, and processing speed [54,55]. Piperazine derivatives (R06AE) were associated with an increased risk of crash, but with a low risk level (median bias-corrected odds ratio of 1.3). Like medicines grouped as “other antihistamines for systemic use drugs” (R06AX), they are prescribed for the treatment of allergic rhinitis and urticaria and are mostly second-generation antihistamines. However, cetirizine and levocetirizine (R06AE) were found to be associated with a higher risk of sedation than loratadine (R06AX) [56,57].

Among medicines showing risk associations with a less consistent pattern in terms of the length of the washout period, benzodiazepine derivatives (N05BA), benzodiazepine-related drugs (N05CF), and plain ACE inhibitors (C09AA) were 3 medicines inconsistently but frequently selected among the 90 models. Moreover, they were among the 10 most consumed medicines. The association between the use of benzodiazepines and the risk of road traffic crashes has been documented with consistent results among drivers. Benzodiazepines have also been shown to be associated with an increased risk of falling in the elderly, which may be due to attention, gait, and balance impairment [58], effects that might also increase the risk of crash for pedestrians. One study has reported a significantly higher risk of at-fault crash involvement with the use of ACE inhibitors in the elderly, medicines commonly used to treat hypertension [21].

Results for 3 medicines were unexpected: plain antiandrogens (G03HA), amino acids (B02AA), and gonadotropin-releasing hormone analogues (L02AE) showed a strong association with the risk of pedestrian crash. However, their levels of use in our sample of injured pedestrians were all under 0.3%, and their estimated odds ratios were based on a small number of discordant pairs. In addition, these medicines were all attributed a level 1 warning pictogram, which means that their risk is low, heterogeneous, and strongly dependent on individuals’ pharmacodynamic parameters.

In summary, our results suggest that several classes of medicine are associated with an increased risk of pedestrian crash. Improving awareness of this risk is, therefore, a necessity, as the risks of medicines in road safety have hitherto been thought to concern drivers only. Most of the crashes occurred when the pedestrian was crossing the street, which requires certain levels of physical ability (e.g., walking speed, head mobility), perception (e.g., vision, hearing), and cognitive processing (e.g., attention, processing speed) [59]. Despite an association between medicine use and pedestrian crashes, it is not recommended to drive instead.

## Supporting information

S1 STROBE Checklist Strengthening the Reporting of Observational Studies in Epidemiology (STROBE) checklist. (DOC)

S1 Table Comparison between included and excluded pedestrians. (DOCX)

S1 Text Study pre-analysis plan. (DOCX)

## Abbreviation

ATC: Anatomical Therapeutic Chemical
