# Supplementary material for: Prescription medicine use by pedestrians and the risk of injurious road traffic crashes: A case-crossover study
Source: PLoS Med. 2017 Jul 18;14(7):e1002347. doi: 10.1371/journal.pmed.1002347 (PMC5515401; doi:10.1371/journal.pmed.1002347)
Supplement: S1 STROBE Checklist — (DOC) [file pmed.1002347.s001.doc]

STROBE Statement—Checklist of items that should be included in reports of ***case-control studies***

|  | Item No | | | Recommendation |  |  |
| --- | --- | --- | --- | --- | --- | --- |
| **Title and abstract** | 1 | | | (*a*) Indicate the study’s design with a commonly used term in the title or the abstract | Title | Prescription Medicine use by Pedestrians and the Risk of Injurious Road Traffic Crashes: A Case-Crossover Study. |
|  |  |  |  | (*b*) Provide in the abstract an informative and balanced summary of what was done and what was found | Abstract | See Methods and findings in abstract. |
| Introduction | | | | |  |  |
| Background/rationale | | 2 | | Explain the scientific background and rationale for the investigation being reported | Introduction, paragraph 2. | “Because medicines have the potential to impair the skills needed to perform road users' tasks safely, the association between the use of medicines and the risk of road traffic crash has been studied among vehicle drivers.” |
|  |  |  |  |  | Introduction, paragraph 3 | “Very few studies have investigated the association between the use of medicinal drugs and the risk of road traffic crashes involving pedestrians, and available data remain descriptive, with no comparison group.” |
| Objectives | | 3 | | State specific objectives, including any prespecified hypotheses | Introduction, paragraph 4 | “The aim of this study was to investigate the association between the use of medicines and the increased risk of road traffic crash in pedestrians.” |
| Methods | | | | |  |  |
| Study design | | | 4 | Present key elements of study design early in the paper | Methods, paragraph 1 | “We extracted and matched data from three French nationwide databases…” |
| Setting | | | 5 | Describe the setting, locations, and relevant dates, including periods of recruitment, exposure, follow-up, and data collection | Methods, paragraph 3. | “All police reports available from 1 July 2005 to 31 December 2011 were compiled.” |
|  |  |  |  |  | Methods, paragraph,9 | “Medication exposure was defined as starting one day after dispensing, and exposure duration was estimated from median values reported in a survey on medicine prescription in France” |
| Participants | | | 6 | (*a*) Give the eligibility criteria, and the sources and methods of case ascertainment and control selection. Give the rationale for the choice of cases and controls | Methods, paragraph 6 | “A pedestrian was excluded if the police report did not contain his or her national ID number or if the extraction procedure failed or a link could not be established with the corresponding record in the police national database of injurious crashes.” |
|  |  |  |  |  | Methods, paragraph 10-11 | Case-crossover Analysis section in method. |
|  |  |  |  | (*b*) For matched studies, give matching criteria and the number of controls per case | Methods, paragraph 11 | Case-crossover Analysis section in method. |
| Variables | | | 7 | Clearly define all outcomes, exposures, predictors, potential confounders, and effect modifiers. Give diagnostic criteria, if applicable | Methods, paragraph 3 | “An injurious crash is defined as a crash occurring in a road open to public circulation, involving at least one vehicle and resulting in at least one victim needing medical attention or being killed.” |
|  |  |  |  |  | Methods, paragraph 8 | “For the present study, only prescription medicines ranking from levels 1 to 3 were included in the analysis.” |
| Data sources/ measurement | | | 8* | For each variable of interest, give sources of data and details of methods of assessment (measurement). Describe comparability of assessment methods if there is more than one group | Methods, paragraph 5 | “The healthcare insurance database covers the entire population of France. A record is added each time a reimbursed prescription medicine is dispensed to an outpatient at a pharmacy; the record includes national ID number, date of dispensing, and the seven-digit code that identifies medicines.” |
| Bias | | | 9 | Describe any efforts to address potential sources of bias | Methods, paragraph 9 | “To ensure that the medicines were not prescribed as a consequence of injuries sustained in the crash, medicines dispensed on the day of the crash were not considered in the analysis.” |
|  |  |  |  |  | Methods, paragraph 13 | ”To correct bias in the estimated coefficients, we fitted the unpenalized logistic regression model with the exposures retained in the model (those having a nonzero point estimate of log-odds ratio)” |
| Study size | | | 10 | Explain how the study size was arrived at | NA |  |
| Quantitative variables | | | 11 | Explain how quantitative variables were handled in the analyses. If applicable, describe which groupings were chosen and why | NA |  |
| Statistical methods | | | 12 | (*a*) Describe all statistical methods, including those used to control for confounding | Methods, paragraph 10-14 | See Case-crossover Analysis and Least Absolute Shrinkage and Selection Operator (Lasso) Analysis section of the Method. |
|  |  |  |  | (*b*) Describe any methods used to examine subgroups and interactions | NA |  |
|  |  |  |  | (*c*) Explain how missing data were addressed | NA |  |
|  |  |  |  | (*d*) If applicable, explain how matching of cases and controls was addressed | Methods, paragraph 10 | “The exposure frequency during a period just before the crash (case period) is compared with the exposure frequency during an earlier period (control period) in the same subject” |
|  |  |  |  | (*e*) Describe any sensitivity analyses | NA |  |
| Results | | | | |  |  |
| Participants | | | 13* | (a) Report numbers of individuals at each stage of study—eg numbers potentially eligible, examined for eligibility, confirmed eligible, included in the study, completing follow-up, and analysed | Results, fig 2 | Fig 2 |
|  |  |  |  | (b) Give reasons for non-participation at each stage | Results, paragraph 1 | “Among these, 186 636 (86.8%) were matched with a corresponding record in the police national database of injurious crashes. The linkage failed for national ID numbers corresponding to road users involved in a crash but not captured in the police national injurious crashes database and for individuals not involved in the crash (e.g. witnesses).” |
|  |  |  |  | (c) Consider use of a flow diagram | Results, fig 2 | Fig 2 |
| Descriptive data | | | 14* | (a) Give characteristics of study participants (eg demographic, clinical, social) and information on exposures and potential confounders | Results, table 1 | Table 1 |
|  |  |  |  | (b) Indicate number of participants with missing data for each variable of interest | Results, table 1 | Table 1 |
| Outcome data | | | 15* | Report numbers in each exposure category, or summary measures of exposure | Results, table 1 | Table 1 |
| Main results | | | 16 | (*a*) Give unadjusted estimates and, if applicable, confounder-adjusted estimates and their precision (eg, 95% confidence interval). Make clear which confounders were adjusted for and why they were included | Results, table 2 | Table 2 |
|  |  |  |  | (*b*) Report category boundaries when continuous variables were categorized | NA |  |
|  |  |  |  | (*c*) If relevant, consider translating estimates of relative risk into absolute risk for a meaningful time period | NA |  |
| Other analyses | 17 | | Report other analyses done—eg analyses of subgroups and interactions, and sensitivity analyses | | NA |  |
| Discussion | | | | |  |  |
| Key results | 18 | | Summarise key results with reference to study objectives | | Discussion, paragraph 1 | “The analysis of 16 458 pedestrians involved in a road crash between 1^st^ July 2005 and 31^st^ December 2011, among which about 5000 were included in the analyses, identified 48 medicine classes associated with an increased risk for pedestrians of being involved in an injurious road traffic crash. Among them, the 10 most consumed medicines included benzodiazepines and benzodiazepine-related drugs, antihistamines, anti-inflammatory and antirheumatic drugs.” |
| Limitations | 19 | | Discuss limitations of the study, taking into account sources of potential bias or imprecision. Discuss both direction and magnitude of any potential bias | | Discussion, paragraph 2 | “Injury severity and age were associated with the probability of being part of the study. |
|  |  |  |  |  | Discussion, paragraph 3 | “[…] there is a lack of information about self-medication and the use of over-the-counter drugs” |
|  |  |  |  |  | Discussion, paragraph 4 | “In studies on medicines, both the treatment and the condition for which the treatment was prescribed can be associated with the outcome of interest.”  “Confounding may also remain for other time-varying factors such as increased enforcement of speeding laws.”  “[…] no information was available on how much the participants walked during the control periods.” |
|  |  |  |  |  | Discussion, paragraph 5 | “Analyses were not adjusted for alcohol consumption because the information was not available during control periods.” |
| Interpretation | 20 | | Give a cautious overall interpretation of results considering objectives, limitations, multiplicity of analyses, results from similar studies, and other relevant evidence | | Discussion, paragraph 10 | In summary, our results suggest that several classes of medicine are associated with an increased risk of pedestrian crash. Improving awareness of this risk is, therefore, a necessity, as the risks of medicines in road safety have hitherto been thought to concern drivers only. Most of the crashes occurred when the pedestrian was crossing the street, which requires certain levels of physical ability (e.g. walking speed, head mobility), perception (e.g. vision, hearing), and cognitive processing (e.g. attention, processing speed).[59] Despite an association between medicine use and pedestrian crashes, it is not recommended to drive instead. |
| Generalisability | 21 | | Discuss the generalisability (external validity) of the study results | | NA |  |
| Other information | | | | |  |  |
| Funding | 22 | | Give the source of funding and the role of the funders for the present study and, if applicable, for the original study on which the present article is based | |  | “The CESIR-A project was supported by the French Direction de la sécurité et de la circulation routières (DSCR) and the French National Institute of Health and Medical Research (Equipe INSERM). The present study is part of the Drugs Systematized Assessment in real-liFe Environment (DRUGS-SAFE) research program funded by the French Medicines Agency (Agence Nationale de Sécurité du Médicament et des Produits de Santé, ANSM); grants received for the year 2015: 900,000 euros. This program aims at providing an integrated system allowing the concomitant monitoring of drug use and safety in France. The potential impact of drugs (e.g. psychotropics), frailty of populations and seriousness of risks drive the research program. This publication represents the views of the authors and does not necessarily represent the opinion of the French National Agency for Medicines and Health Products Safety (ANSM).” |

*Give information separately for cases and controls.
