# Supplementary material for: Prescription medicine use by pedestrians and the risk of injurious road traffic crashes: A case-crossover study
Source: PLoS Med. 2017 Jul 18;14(7):e1002347. doi: 10.1371/journal.pmed.1002347 (PMC5515401; doi:10.1371/journal.pmed.1002347)
Supplement: S1 Table — (DOCX) [file pmed.1002347.s002.docx]

**S1 Table:** Comparison between included and excluded pedestrians

|  |  | No. (%) Included | No. (%) not Included |
| --- | --- | --- | --- |
| Total |  | 16458 | 69850 |
| Gender** | Female | 9655 (58.7) | 36090 (51.7) |
| Age (years)** | <18 | 1455 (8.86) | 21093 (30.23) |
|  | 18-24 | 1641 (9.99) | 7210 (10.33) |
|  | 25-34 | 1731 (10.53) | 6808 (9.76) |
|  | 35-44 | 1810 (11.02) | 6299 (9.03) |
|  | 45-54 | 2122 (12.91) | 6526 (9.35) |
|  | 55-64 | 2255 (13.72) | 6615 (9.48) |
|  | 65-74 | 2022 (12.31) | 5677 (8.14) |
|  | ≥ 75 | 3395 (20.66) | 9552 (13.69) |
| Responsible (as determined by police force)** |  | 2369 (14.40) | 12710 (18.20) |
| Injury severity** | Unhurt | 123 (0.75) | 1589 (2.27) |
|  | Slightly injured | 8197 (49.81) | 40710 (58.28) |
|  | Seriously injured | 7741 (47.03) | 24490 (35.06) |
|  | Killed | 397 (2.41) | 3061 (4.38) |
| Weather** | Normal | 12844 (78.05) | 56841 (81.38) |
|  | Light rain | 1852 (11.25) | 7076 (10.13) |
|  | Heavy rain | 473 (2.87) | 1512 (2.16) |
|  | Snow-hail | 118 (0.72) | 429 (0.61) |
|  | Fog-smoke | 76 (0.46) | 214 (0.31) |
|  | Strong wind-storm | 34 (0.21) | 109 (0.16) |
|  | Blinding weather | 381 (2.32) | 895 (1.28) |
|  | Cloudy | 572 (3.48) | 2348 (3.36) |
|  | Other | 107 (0.65) | 425 (0.61) |
| Season** | Spring | 3289 (19.98) | 16187 (23.17) |
|  | Summer | 3475 (21.11) | 15589 (22.32) |
|  | Autumn | 5601 (34.03) | 22187 (31.76) |
|  | Winter | 4093 (24.87) | 15887 (22.74) |
| Time of day** | 05:00-10:59 | 4464 (27.12) | 15730 (22.52) |
|  | 11:00-13:59 | 2865 (17.41) | 12459 (17.84) |
|  | 14:00-19:59 | 7762 (47.16) | 33924 (48.57) |
|  | 20:00-22:59 | 923 (5.61) | 4912 (7.03) |
|  | 23:00-01:59 | 286 (1.74) | 1832 (2.62) |
|  | 02:00-04:59 | 158 (0.96) | 993 (1.42) |
| Crash day** | Weekdays | 13500 (82.03) | 56011 (80.19) |
|  | Saturday | 1958 (11.90) | 8477 (12.14) |
|  | Sunday | 1000 (6.08) | 5362 (7.68) |
| Lighting* | Daylight | 12028 (73.08) | 51264 (73.39) |
|  | Dawn or dusk | 1007 (6.12) | 3839 (5.50) |
|  | Dark, no street lights | 498 (3.03) | 2163 (3.10) |
|  | Dark, street lights off | 132 (0.80) | 519 (0.74) |
|  | Dark, street lights on | 2793 (16.97) | 12065 (17.27) |
| Pedestrian’s action** | Not reported | 715 (4.34) | 3381 (4.84) |
|  | Moving | 1340 (8.14) | 5086 (7.28) |
|  | Crossing | 12474 (75.79) | 50856 (72.81) |
|  | Playing-running | 470 (2.86) | 4559 (6.53) |
|  | Other | 1459 (8.86) | 5968 (8.54) |
| Pedestrian’s location** | Not reported | 1632 (9.92) | 7606 (10.89) |
|  | More than 50 meters from a crosswalk | 2064 (12.54) | 10378 (14.86) |
|  | Less than 50 meters from a crosswalk | 3416 (20.76) | 16822 (24.08) |
|  | Crosswalk without a traffic light | 2990 (18.17) | 9987 (14.30) |
|  | Crosswalk with a traffic light | 4833 (29.37) | 18895 (27.05) |
|  | Sidewalk | 1028 (6.25) | 4270 (6.11) |
|  | Other | 495 (3.01) | 1892 (2.71) |

*p<0.05.

**p<0.0001.
