# Supplementary material for: Prescription medicine use by pedestrians and the risk of injurious road traffic crashes: A case-crossover study
Source: PLoS Med. 2017 Jul 18;14(7):e1002347. doi: 10.1371/journal.pmed.1002347 (PMC5515401; doi:10.1371/journal.pmed.1002347)
Supplement: S1 Text — (DOCX) [file pmed.1002347.s003.docx]

Supplementary information _ Study Analysis Plan

1. **Hypothesis**

Some medicines that could affect the ability to drive have been identified. We assume that some of these medicines may also be associated with an increased risk of injurious road traffic crash in pedestrians.

1. **Method**

An exploratory study of the prescription medicines ranking from level 1 to 3 (levels of the French classification of medicines according to driving impairment risk) and the risk of road traffic crash in pedestrians.

Descriptive analysis

Descriptive analysis will consist in:

- Description of the study population: pedestrian characteristics (age, gender, responsibility attributed by police forces, injury severity and pedestrian’s action and location) and crash characteristics (weather, season, time and day of the crash and lighting).
- Study of the characteristics (same variables) associated with the probability of being part of the study using logistic regression model.

Study design

Case-crossover designs will be implemented with one control period per case. The control period will be moved from 30 to 119 days before the crash day, leading to 90 case-crossover designs.

Multivariate analysis

The bootstrap-enhanced least absolute shrinkage operator (Bolasso) method will be used to study the association between medicine prescription and the risk of road traffic crash in pedestrians.

Two parameters will have to be tuned:

- The optimal amount of shrinkage will be estimated by cross-validation.
- The frequency threshold will be determined using the Akaike criterion over 1000 bootstrap samples.

For each control period and at the end of the 1000 bootstraps, the unpenalized logistic regression model with the exposures retained in the model (those having a nonzero point estimate of log-odds ratio) will be fitted to correct bias in the estimated coefficients.

Results for each of the 90 control periods will be presented in a figure to investigate whether patterns can be found according to the location of the control period. In addition, a table will summarize, for each medicine, the number of case-crossover designs in which the exposure was selected, the median and interquartile range of the bias-corrected odds ratio, and the median and interquartile range of the number pairs with unequal exposures.

1. **Variation to the analysis plan**

Overall, analyses were performed as initially planned.

Given the number of exposure variables, a table of the 10 most consumed medicines among the study population was added in order to enrich the interpretation and discussion of the results.
